# Bone marrow-derived progenitor cells in end-stage lung disease patients

**DOI:** 10.1186/1471-2466-13-48

**Published:** 2013-08-03

**Authors:** Sarah E Gilpin, Kalvin Lung, Geoffrey T de Couto, Marcelo Cypel, Masaaki Sato, Lianne G Singer, Shaf Keshavjee, Thomas K Waddell

**Affiliations:** 1 Latner Thoracic Surgery Research Laboratories, Division of Thoracic Surgery, Toronto General Hospital, University Health Network, University of Toronto, North Wing, 9N - 949, 200 Elizabeth Street, Toronto, ON M5G 2C4, Canada; 2 Division of Cardiology, Heart and Stroke/Richard Lewar Centre of Excellence, University Health Network, University of Toronto, Toronto, Ontario, Canada

**Keywords:** Fibrocytes, Clara cell, Lung progenitor, Migration

## Abstract

**Background:**

Chronic lung diseases are marked by progressive inflammation, tissue damage and remodelling. Bone marrow-derived progenitor cells may contribute to these processes. The objectives of this study were to (1) to quantify CD45^+^Collagen-1^+^ fibrocytes and a novel epithelial-like population of bone marrow-derived cells, which express Clara Cell Secretory Protein, in patients at the time of lung transplant and (2) to evaluate mediators that may act to recruit these cells during injury.

**Methods:**

Using an observational design, progenitor cells were quantified by flow cytometry from both bone marrow (BM) and peripheral blood (PB). Migration was tested using *in vitro* transwell assays. Multiplex bead-based assays were used to quantify plasma cytokines.

**Results:**

An increase in CD45^+^Collagen-1^+^ fibrocytes was found in pulmonary fibrosis and bronchiolitis obliterans patients. Cystic fibrosis patients had an increase in CCSP^+^ cells in both the BM and PB. The proportion of CCSP^+^ cells in the BM and PB was correlated. CCSP^+^ cells express the chemokine receptors CCR2, CCR4, CXCR3, and CXCR4, and significantly migrated *in vitro* toward Stromal Derived Factor-1 (SDF-1) and Stem Cell Growth Factor-β (SCGF-β). Plasma cytokine levels differed between disease groups, with a significant correlation between SCGF-β and CCSP^+^ cells and between Monocyte Chemotactic Protein-1 and fibrocytes.

**Conclusions:**

Different bone marrow-derived cells are found in various lung diseases. Increased fibrocytes were associated with fibrotic lung diseases. An increase in the novel CCSP^+^ epithelial-like progenitors in cystic fibrosis patients was found. These differences may be mediated by alterations in plasma cytokines responsible for cell recruitment.

## Background

Many pulmonary pathologies including cystic fibrosis (CF), pulmonary fibrosis (PF), chronic obstructive pulmonary disease (COPD), and pulmonary hypertension (PH) follow a progressive course, and at their end stages are treatable only by transplantation [1]. Taken together, features of chronic lung diseases commonly include excessive inflammation, tissue remodelling, and epithelial damage, which ultimately leads to a loss of function and organ failure [2,3].

The contribution of bone marrow-derived cell populations to adult tissue repair has been widely studied, and although controversial, evidence exists implicating various progenitor populations in both tissue remodelling, pathogenic fibrosis, and productive repair. Numerous investigators have described therapeutic benefits with exogenously applied marrow-derived populations [4-6], however the endogenous role of such populations is uncertain [7]. In the context of human chronic lung disease, we chose to investigate two bone marrow-derived populations to determine the numbers of these cells in various disease states.

We have previously described a novel epithelial-like progenitor population marked by Clara Cell Secretory Protein (CCSP) found within the bone marrow (BM) and peripheral blood (PB) [8]. In a model of naphthalene-induced lung injury in mice, a transient increase in bone marrow and peripheral blood CCSP^+^ cells was measured following injury. In addition, when labelled CCSP^+^ cells were delivered (trans-tracheally or intravenously), injured murine lungs were found to were found preferentially retain CCSP^+^ as compared to CCSP^-^ cells. For these reasons, it was hypothesized that CCSP^+^ epithelial-like progenitor cells may also be important in human lung disease.

In addition, circulating CD45^+^Collagen-1^+^ fibrocytes have also been implicated in the development of tissue fibrosis, in both animal models and human disease. Inhibition of this cell population though blockage of SDF-1/CXCR4-mediated migration has been shown to attenuate bleomycin-induced lung injury in mice [9]. Quantification of circulating fibrocyte numbers has also been shown to be an independent predictor of survival in pulmonary fibrosis patients [10], and we recently reported an increase in this population in patients with bronchiolitis obliterans [11].

These two populations have not previously been compared across disease groups and taken together may play an important role in disease pathology. This study aimed to quantify both cell populations in the bone marrow and/or peripheral blood of end-stage lung disease patient at the time of transplantation. We hypothesise that a specific relationship may exist between the number and recruitment ability of specific bone marrow-derived cell populations and specific end-stage lung disease pathologies. Utilizing an observational study design we aimed to investigate these relationships across various end-stage lung diseases. Investigation of plasma cytokine mediators of cell mobilization or trafficking also aimed to elucidate key differences in these factors between disease groups and in relation to progenitor cell numbers.

## Methods

The study was designed with a cross-sectional, observational approach and was approved by the University Health Network Research Ethics Board (#07-0598TE). Written informed consent was obtained from all subjects. The study population consisted of lung transplant recipients at the Toronto General Hospital between Nov 2007 and Jan 2011. Lung donors were also included as a comparison group.

### Sample preparation

Bone marrow (BM) was obtained from the exposed sternum and prepared by Ficoll. Equal parts heparinized peripheral blood (10 ml) were prepared by Ficoll isolation for peripheral blood mononuclear cells (PBMCs), which was used for CCSP cell quantification, and by high-speed centrifugation for peripheral blood leukocytes (PBLs), which was used for fibrocyte quantification. PBLs were treated with red cell lysis buffer. Plasma was collected from the centrifuged aliquot.

### Flow cytometry

For CCSP^+^ cell quantification, freshly isolated BMCs and PBMCs were blocked with 10% goat serum and 10% Fc Block (Miltenyi Biotech), stained with rabbit anti-mouse/human CCSP (1:200; Upstate Labs) or IgG control antibody (R&D), followed by Alexa Fluor 488 secondary (1:1000; Invitrogen).

For fibrocyte quantification, freshly isolated PBLs we blocked as above, stained with mouse anti-human CD45 (1:5: PerCP-conjugated, BD Biosciences), permeabilizied using Cytoperm solution (BD Biosciences), and subsequently stained with rabbit-anti-human collagen type-1 (1:100; Rockland Immunochemicals) or IgG control antibody, followed by AlexaFluor 488 secondary (1:1000; Invitrogen). For double-staining experiments, CCSP antibody was detected with an AlexaFluor 647 secondary (Invitrogen) and the chemokine receptors detected with PE-conjugated antibodies (BD Biosciences), providing sufficient spectral emission separation.

All data was generated using a Coulter Cytomics FC500 analyzer, collecting 20,000 events per sample, and analyzed with FlowJo software. Sorting of isolated CCSP^+^ peripheral bloods cells for PCR was performed on a BD FACSAria II, starting with 60 ml of peripheral blood from a healthy, male volunteer.

### Real-time PCR

Real-time quantitative PCR was performed by Taqman technology (Applied Biosystems). In brief, total RNA was isolated using the RNeasy Kit (Qiagen) and RNA concentrations were determined by Nanodrop analyzer (Thermo Scientific). First-strand cDNA was generated using Superscript III (Sigma) protocol. Real-time PCR was performed for amplification of the CCSP or Collagen-1 gene products (Taqman probes Hs00171092_m1 and Hs00164004_m1). Beta-2-Microglobulin was used as endogenous control (Applied Biosystems; 4333766 T). Human bronchus tissue (positive control) was collected from explanted recipient lungs, subject to collagenase digestion (Stem Cell Technologies), and prepared in parallel. Control RNA samples not subjected to reverse transcription (NRT) and water (no template, NTC) were used as negative controls.

### *In vitro* transwell migration assays

Migration of CCSP^+^ cells was assessed in response to chemotactic stimuli in healthy subjects (median age = 29 yrs, M:F = 6:3) vs. transplant recipients (median age = 45.5, M:F = 9:7). Initially, 1 × 10^6^ BMCs or PBMCs were layered onto a 5 μm-pore membrane insert and placed into contact with DMEM + 10% FBS + the cytokine Regulated upon Activation, Normal T-cell Expressed, and Secreted (RANTES) 20 ng/ml, Interferon gamma-induced Protein 10 (IP-10) 25 ng/ml, Stromal-Derived Factor −1 (SDF-1) 10 ng/ml, or Stem Cell Growth Factor-β (SCGF-β) 5 ng/ml; Peprotech) in a 24-well tissue culture plate (Costar). Following 2 hours of migration all cells recovered in the lower chamber were collected, counted, and analyzed for CCSP expression by flow cytometry. Migrated CCSP^+^ cells were determined as follows:

(1) $$\begin{array}{l} \mathrm{Total}\;\mathrm{cells}\;\mathrm{migrated}\;\times \;\%\;\mathrm{CCS}P^{+}\\ \;=\;\mathrm{Absolute}\;\mathrm{CCS}P^{+}\;\mathrm{cells}\;\mathrm{migrated} \end{array}$$

(2) $$\begin{array}{l} \mathrm{Absolute}\;\mathrm{CCS}P^{+}\;\mathrm{migrated}\;\mathrm{to}\;\mathrm{chemokine}/\mathrm{Absolute}\\ \;\mathrm{CCS}P^{+}\;\mathrm{in}\;\mathrm{untreated}\;\mathrm{sample}\;=\;\mathrm{Fold}\;\mathrm{CCS}P^{+}\\ \;\mathrm{migrated} \end{array}$$

### Protein quantification

Plasma samples were analyzed by Luminex-based multiplex array (BioRad Bio-Plex System) according to manufacturer protocols. Targets analyzed are listed in Additional file 1: Table S3. Bio-Plex Manager software was used for data acquisition.

### Statistics

Statistical analysis was performed using GraphPad Prism software. Data are presented as median ± range, with whiskers encompassing the 5-95th percentiles. Normality was tested using the D’Agostino & Pearson omnibus test and non-parametric tests were used throughout. A Mann–Whitney test was used for comparison of non-parametric variables between two groups. Multiple comparisons were made using a Kruskal-Wallis test with Dunn’s multiple comparison post-test correction. Spearman rank test with correlation coefficient was used to determine relationships between two variables. Statistical significance was defined as p < 0.05.

## Results

### Identification of progenitor populations in end-stage lung disease patients

**Figure 1:**
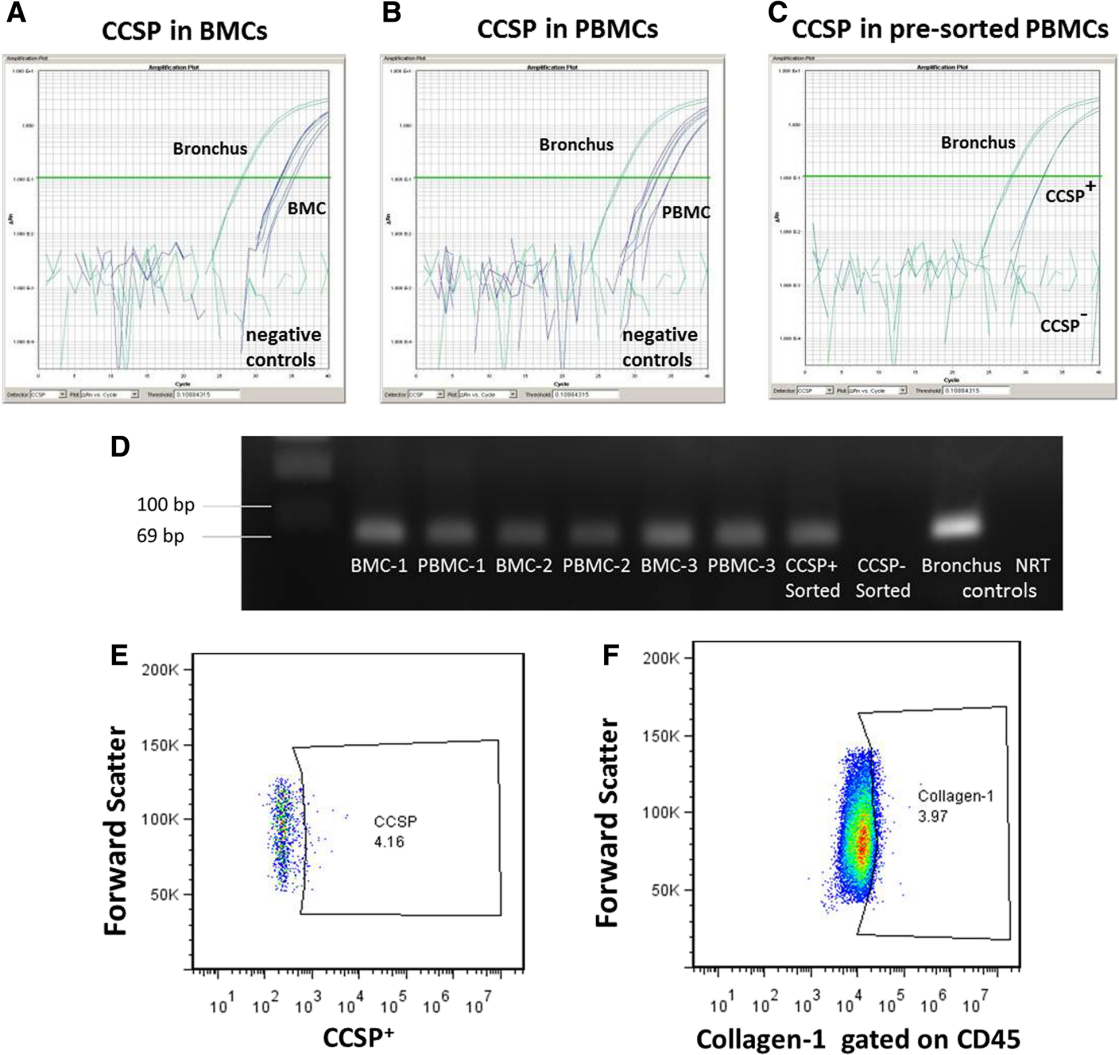
**Identification of progenitor populations.** Taqman PCR measurement of **(A)** Clara Cell Secretory Protein (CCSP) gene expression in human bone marrow cells (BMCs), **(B)** peripheral blood mononuclear cells (PBMCs), and **(C)** PBMCs pre-sorted for CCSP by flow cytometry. No template and no reverse transcription negative controls are included. **(D)** Gel electrophoresis of PCR product after Taqman-based amplification. Positive control (bronchus) and negative no reverse transcription (NRT) controls are included. **(E)** Flow cytometry gating for measurement of CCSP^+^ PBMCs based on isotype control staining. **(F)** Flow cytometry gating for measurement of CD45^+^Collagen-1^+^ peripheral blood leukocytes (PBLs) based on isotype control staining.

Circulating bone marrow-derived cell populations were defined by (1) Clara Cell Secretory Protein (CCSP) expression for epithelial-like progenitors and by (2) dual expression of CD45 and intracellular collagen-1 expression for fibrocytes. To validate the expression of CCSP mRNA in the bone marrow and peripheral blood, Taqman PCR was utilized. CCSP mRNA was detected in human bone marrow cells (BMCs) and peripheral blood mononuclear cells (PBMCs) from 3 randomly selected lung transplant recipients, as well as in control lung bronchus tissue, but absent in experimental controls (no reverse transcription and no template controls) (Figure 1A-B). As a further proof-of-principle, peripheral blood cells from a healthy volunteer were isolated and sorted for CCSP by flow cytometery, representing less than 1% of total PBMCs, and then analyzed by PCR. CCSP mRNA was also detected in the pre-sorted population but not in CCSP-negative sorted cells (Figure 1C). The resulting amplification product was further analyzed by gel electrophoresis to confirm the correct amplicon size (Figure 1D), and compared to positive human bronchus tissue mRNA and a mixture of sample mRNAs not subjected to reverse transcription (NRT) to control for genomic DNA contamination. All subsequent quantification of progenitor cells populations was determined by flow cytometry. A representative plot identifying the positively gated populations based on initial isotype staining of 1% is shown for both CCSP^+^ cells and CD45^+^Collagen-1^+^ fibrocytes (Figure 1E-F). Further details of the gating strategy have been previously published [11].

Bone marrow and peripheral blood samples were collected from patients at the time of lung transplant (n = 154). A summary of relevant lung recipient demographics, including age, gender, diagnosis, diabetes status, BMI, and graft number is presented in Table 1. In addition, blood and bone marrow samples were obtained from lung donors prior to organ recovery (n = 36), the details of which are summarised in Table 2. For logistical reasons, not every patient undergoing lung transplantation or donor procurement could be analyzed in this study. A comparison of recipient and donor demographics from patients included in this analysis compared to the demographics of all those transplanted at our centre in the same time period identified no significant differences in these parameters (Additional file 2: Table S1 and Additional file 3: Table S2).

**Table 1:** Demographics of lung transplant recipients sampled

	**All included transplant**
	**recipients**
	**n = 154**
Recipient	
Age at transplantation (SD), yrs	50.9 ± 14.9
Gender, total (%)	
Male	89 (57.8)
Female	65 (42.2)
Diagnosis	
COPD/Emphysema + Alpha-1 Antitrypsin Deficiency	43 (27.9)
Pulmonary Fibrosis + Scleroderma	52 (33.8)
Cystic Fibrosis + Bronchiectasis	34 (22.1)
PPH + Eisenmenger’s + Congenital Abnormalities	14 (9.1)
Retransplant + BO	6 (3.9)
Other	5 (3.2)
Diabetes mellitus	
Non-Insulin Dependent	17 (11.0)
Insulin Dependent	20 (13.0)
No Diabetes	117 (76.0)
BMI, mean (SD), kg/rn2	23.6 ± 4.8
Graft Number	
First	148 (96.1)
Second	6 (3.9)

**Table 2:** Demographics of lung donors sampled

	**All donors sampled**
	**(n = 36)**
Age at transplantation yrs ± SD	46.7 ± 17.7
Gender, n (%)	
Male	15 (41.7)
Female	21 (58.3)
Cause of Death	
Cerebrovascular/Stroke	21 (58.3)
Anoxia/Hypoxia	2 (5.6)
Head Trauma	7 (19.4)
Spontaneous Intracranial Hemorrhage	3 (8.3)
Primary CNS Tumor	1 (2.8)
Cardiac Arrest/MI	1 (2.8)
Other	1 (2.8)
Diabetes mellitus	
Diabetes	2 (5.6)
No Diabetes	34 (94.4)
BMI, mean (SD, kg/m^2^	25.6 ± 4.9

**Figure 2:**
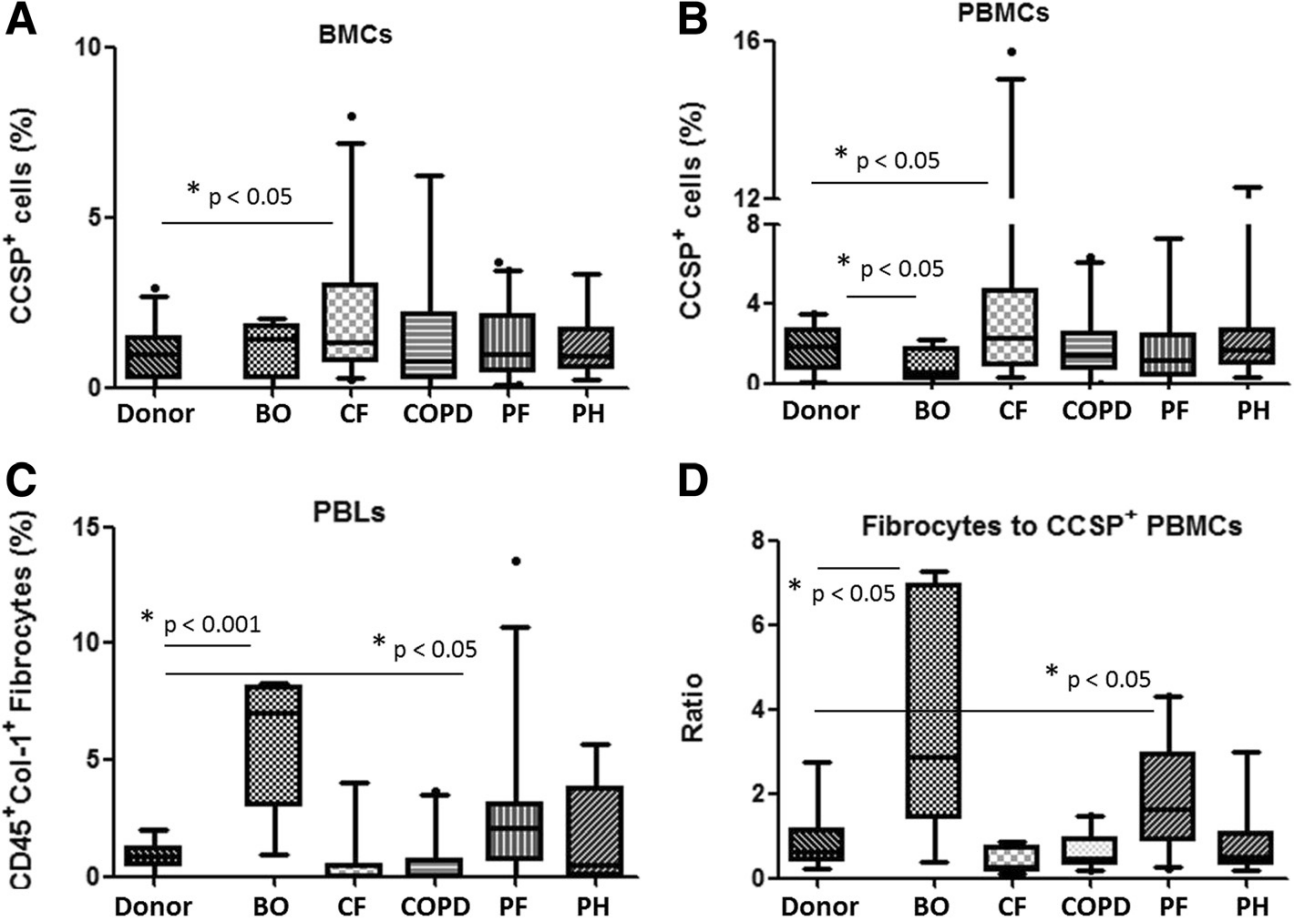
**Differential progenitor cell profiles in end-stage lung disease patients. (A)** Percentage of bone marrow cells (BMCs) positive for CCSP in each disease group (n = 26 donor, n = 5 Bronchiolitis Obliterans (BO), n = 27 Cystic Fibrosis (CF), n = 34 Chronic Obstructive Pulmonary Disease (COPD), n = 41 Pulmonary Fibrosis (PF), n = 11 Pulmonary Hypertension (PH)). **(B)** Percentage of peripheral blood mononuclear cells (PBMCs) positive for CCSP in each disease group. (n = 29 donor, n = 6 BO, n = 33 CF, n = 41 COPD, n = 53 PF, n = 13 PH). **(C)** Percentage of peripheral blood leukocytes (PBLs) positive for CD45 and collagen-1 in each disease group (n = 17 donor, n = 6 BO, n = 14 CF, n = 22 COPD, n = 26 PF, n = 8 PH). **(D)** Ratio of CCSP^+^ PBMCs to CD45^+^collagen-1^+^ fibrocytes in each disease group, compared to lung donors (n = 13 donor, n = 6 BO, n = 10 CF, n = 17 COPD, n = 18 PF, n = 8 PH). Kruskal-Wallis test with Dunn’s multiple comparison post-hoc analysis. Boxes show the median, 25th and 75th percentiles. Whiskers represent the 2.5 and 97.5 percentiles.

No significant relationships were identified when these progenitor cell populations were analysed by age, gender, or BMI (data not shown). There were significant differences in the proportion of bone marrow-derived cells populations based on underlying lung disease. An increase in the proportion of CCSP^+^ cells was found in the bone marrow of CF patients when compared to lung donors (CF median = 1.33%, Donor median = 0.98, p < 0.05) (Figure 2A). We chose to use lung transplant donors as a comparison group as we had access to sternal marrow biopsy material obtained using identical collection techniques, not easily obtained in any other way. Different cell proportions were also found for CCSP^+^ cells in the peripheral blood. Specifically, CF patients had a greater proportion of CCSP^+^ cells when compared to lung donors (2.28% CF vs. 1.90% Donor, p < 0.05) (Figure 1B), while BO patients had a significantly lower proportion of CCSP^+^ PBMCs compared to donors (0.55% vs. 1.90% Donor, p < 0.05). When circulating CD45^+^collagen-1^+^ fibrocytes were compared between disease groups, an increased proportion was found in both BO patients (p < 0.001) and PF (p < 0.05) patients, when compared to Donors (Median BO = 7.02%, Median PF = 2.07%, Median Donors = 0.85%) (Figure 2C). As the changes in cell numbers appeared to reciprocal, the ratio of fibrocytes-to-CCSP^+^ PBMCs was calculated, and a similar pattern was found, where a predominantly fibrotic phenotype is represented in BO and PF, but not in other lung pathologies (Figure 2D).

In order to determine if the proportion of CCSP^+^ cells was reflective of the total number of CCSP^+^ cells, the absolute cell numbers were determined using total leukocyte counts collected from clinical data (10^9^/L). When absolute cell numbers were compared, the differences in progenitor cell numbers between disease groups were still statistically significant (Additional file 4: Figure S1).

**Figure 3:**
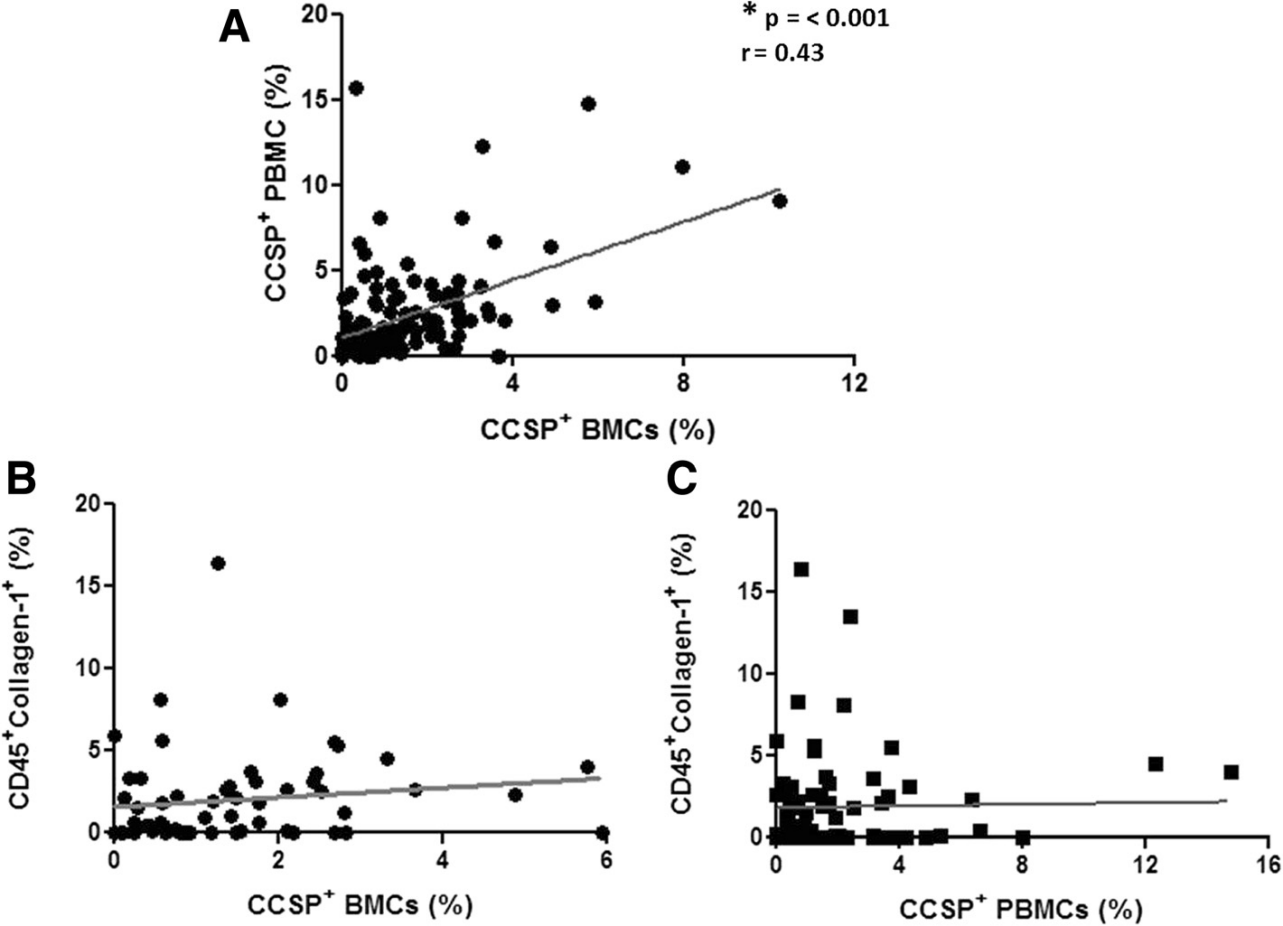
**Relationship between progenitor cell populations. (A)** Correlation between the percentage of Clara Cell Secretory Protein (CCSP^+^) Peripheral Blood Mononuclear Cells (PBMCs) and CCSP^+^ Bone Marrow Cells (BMCs) (n = 119 pairs). **(B)** Lack of correlation between the percentage of CD45^+^Collagen-1^+^ fibrocytes and CCSP^+^ BMCs (n = 59 pairs). **(C)** Lack of correlation between the percentage of CD45^+^Collagen-1^+^ fibrocytes and CCSP^+^ PBMC (n = 74 pairs). Spearman rank test with correlation coefficient.

To investigate the relationship between CCSP^+^ cells within the bone marrow and the proportion in the peripheral blood, data was analyzed including all disease groups together. A significant correlation was found between the number of bone marrow and peripheral blood CCSP^+^ cells (Figure 3A). In contrast, no relationship was found between the number of fibrocytes and either CCSP^+^ BMC or PBMCs (Figure 3B-C).

**Figure 4:**
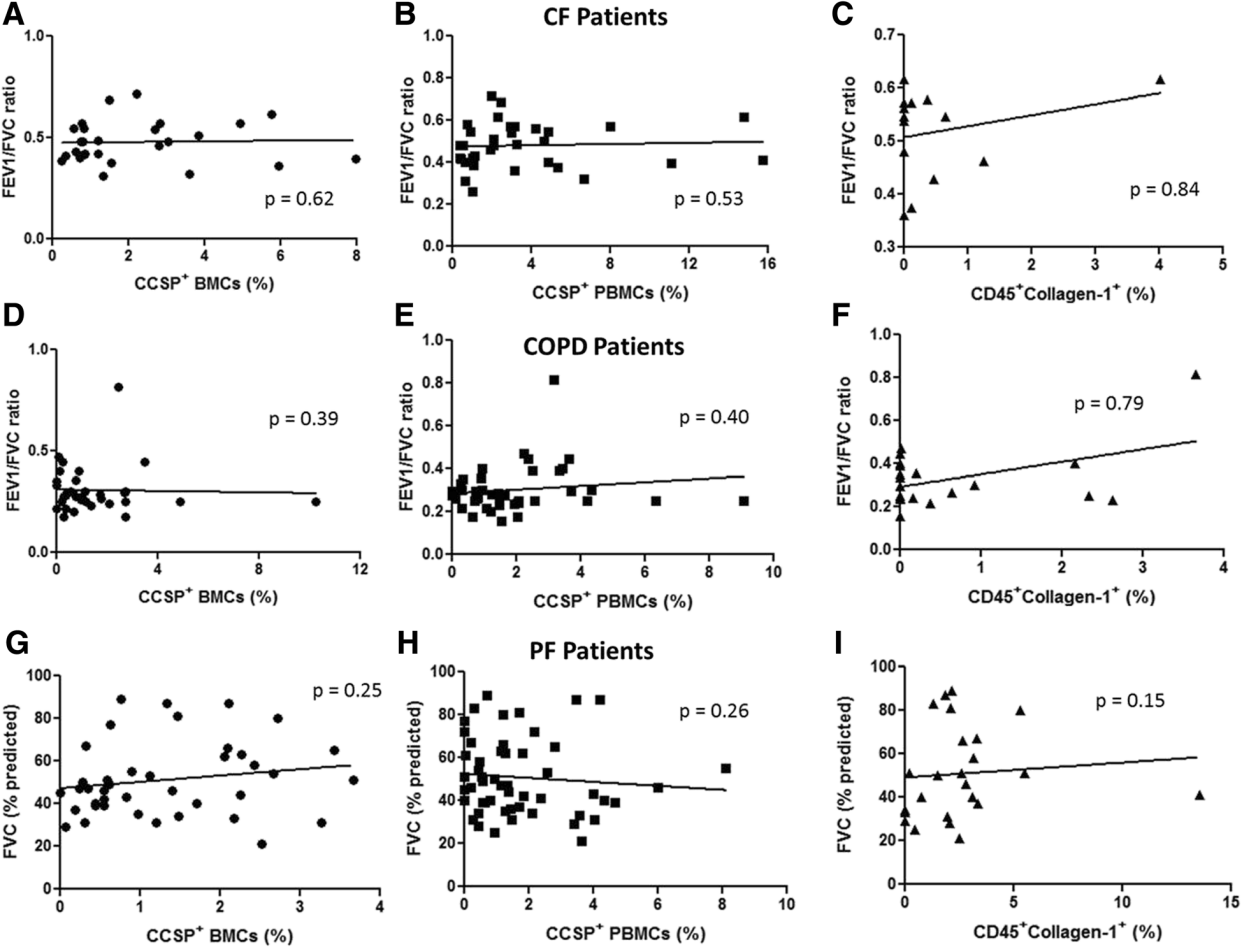
**Lung function and progenitor cell profiles.** No correlation in Cystic Fibrosis (CF) patients between FEV1/FVC and **(A)** Clara Cell Secretory Protein (CCSP+) bone marrow cells (BMCs) (n = 27), **(B)** CCSP + peripheral blood mononuclear cells (PBMCs) (n = 32), or **(C)** CD45 + Collagen-1+ fibrocytes (n = 14). No correlation in Chronic Obstructive Pulmonary Disease (COPD) patients between FEV1/FVC and **(D)** CCSP + BMCs (n = 34), **(E)** CCSP + PBMCs (n = 40), or **(F)** CD45 + Collagen-1+ fibrocytes (n = 22). No correlation in Pulmonary Fibrosis (PF) patients between the measured percentage of predicted of FVC and **(G)** CCSP + BMCs (n = 41), **(H)** CCSP + PBMCs (n = 53), or **(I)** CD45 + Collagen-1+ fibrocytes (n = 26). Spearman rank test.

Analysis of clinical disease indicators relative to progenitor cell numbers was done using spirometric lung function values, using the ratio of the forced expiratory volume in 1 second (FEV1) to forced vital capacity (FVC) (FEV_1_/FVC) ratio for CF and COPD patients, or based on the percentage of predicted FVC for PF patients. No direct relationships were found between lung function measurements when compared with the total number of epithelial-like progenitors in the bone marrow or peripheral blood or with circulating fibrocytes (Figure 4A-I).

In order to further investigate the biology of these cell populations in end-stage lung disease patients, we next analyzed the potential role of receptor-mediated cytokine-induced migration of CCSP^+^ cells.

### Mechanisms of CCSP^+^ progenitor cell recruitment

**Figure 5:**
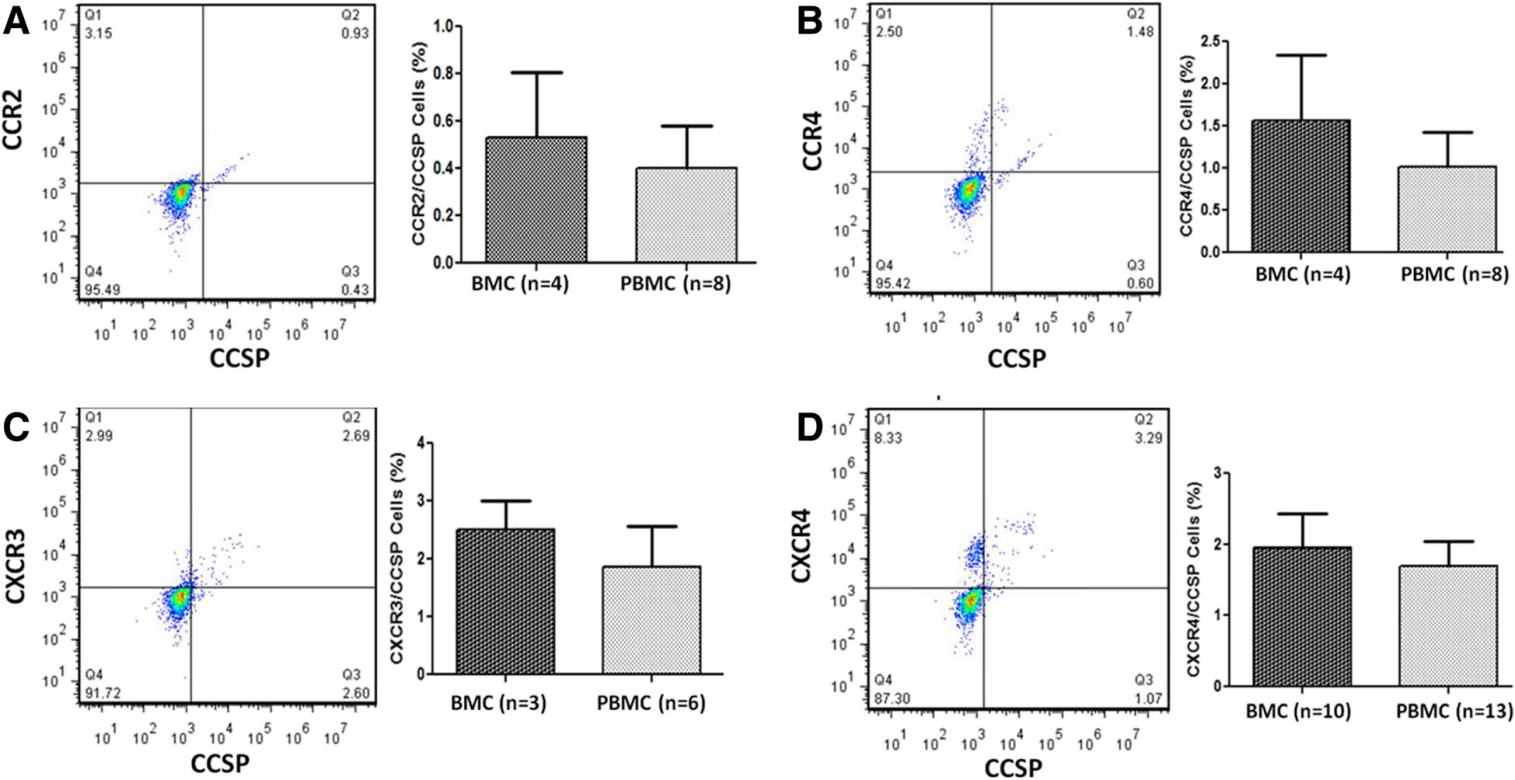
**Chemokine receptor expression by Clara Cell Secretory Protein (CCSP**^**+**^**) Cells.** Dual expression of chemokine receptors and Clara Cell Secretory Protein (CCSP) on bone marrow cells (BMCs) and peripheral blood mononuclear cells (PBMCs). Representative flow plots are based on PBMCs. **(A)** CCR2 **(B)** CCR4 **(C)** CXCR3 **(D)** CXCR4. Bars graphs display the mean and standard error.

In order to explore possible mechanisms of recruitment of these cells chemokine receptor expression was investigated for several chemokines implicated in the literature. Chemokine receptor expression by CCSP^+^ epithelial-like progenitor cells was first examined by flow cytometry. Sub-populations of CCSP^+^ BMC and PBMCs were identified that co-express CCR2, CCR4, CXCR3, or CXCR4 (Figure 5A-D).

**Figure 6:**
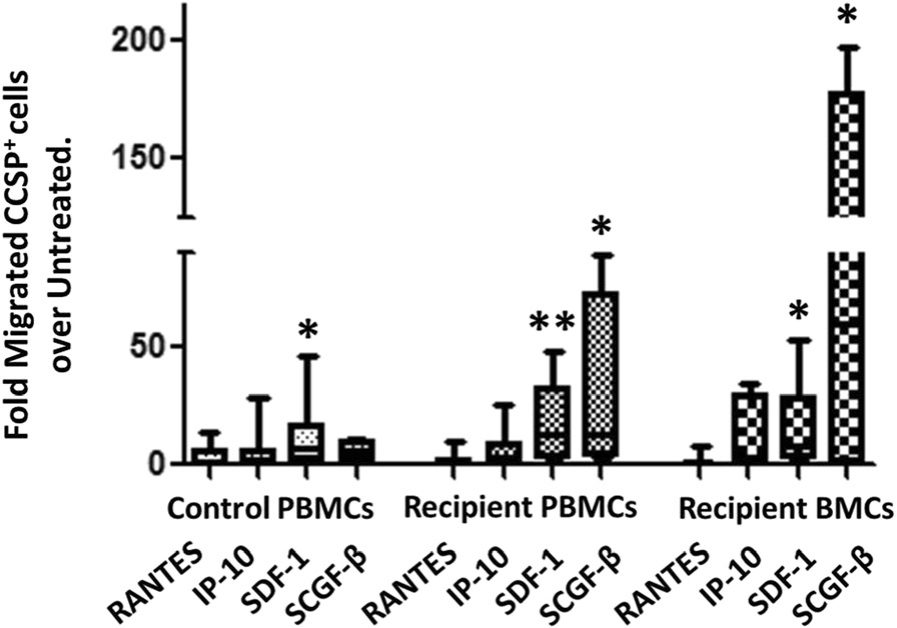
***In vitro *migration assay.** Migration of freshly isolated peripheral blood mononuclear cells (PBMCs) or bone marrow cells (BMCs) in response to chemotactic stimuli Regulated upon Activation, Normal T-cell Expressed, and Secreted (RANTES) (n = 9 control, 11 recipient PBMC, 7 BMC), Interferon gamma-induced protein 10 (IP-10) (n = 9 control, 11 recipient PBMC, 7 BMC), Stromal Derived Factor-1 (SDF-1) (n = 9 control, 13 recipient PBMC, 9 BMC), or Stem Cell Growth Factor-beta (SCGF-β) (n = 4 control, 4 recipient PBMC, 4 BMC), compared to untreated cells. Migrated cells were analyzed for CCSP^+^ expression and normalized to total CCSP^+^ cells in the starting sample. Kruskal-Wallis test with Dunn’s multiple comparison post-hoc analysis, with significance tested against untreated samples (* = p < 0.05, ** = p < .01). Boxes show the median, 25th and 75th percentiles. Whiskers represent the 2.5 and 97.5 percentiles.

To further investigate the ability of CCSP^+^ cells to migrate in response to chemotactic mediators, *in vitro* transwell assays were utilized. Migration of bone marrow or peripheral blood cells (BMCs) freshly isolated from end-stage lung disease patients was investigated in response to the chemotactic stimuli RANTES, IP-10, SDF-1, or SCGF-β and compared to untreated cells (Figure 6). A significant migratory response of CCSP^+^ cells toward SDF-1 was identified for CCSP^+^ PBMCs from control and lung recipient samples, as well as from BMCs from lung recipients, compared to untreated cells in the absence of any chemotactic stimuli. In addition, significant migration in response to SCGF-β was also found for CCSP^+^ BMCs and PBMCs isolated from end-stage lung disease patients (p < 0.05), while no significant migratory response was found for CCSP^+^ PBMCs isolated from healthy controls (Figure 6).

To search for other potentially important cell recruitment mediators, a multiplex array was performed on a subset of end-stage lung disease patients’ plasma. A total of 17 targets were selected based on biological action and quantified simultaneously (see methods and Additional file 1: Table S3). These results were then analyzed in relation to progenitor cell numbers.

**Figure 7:**
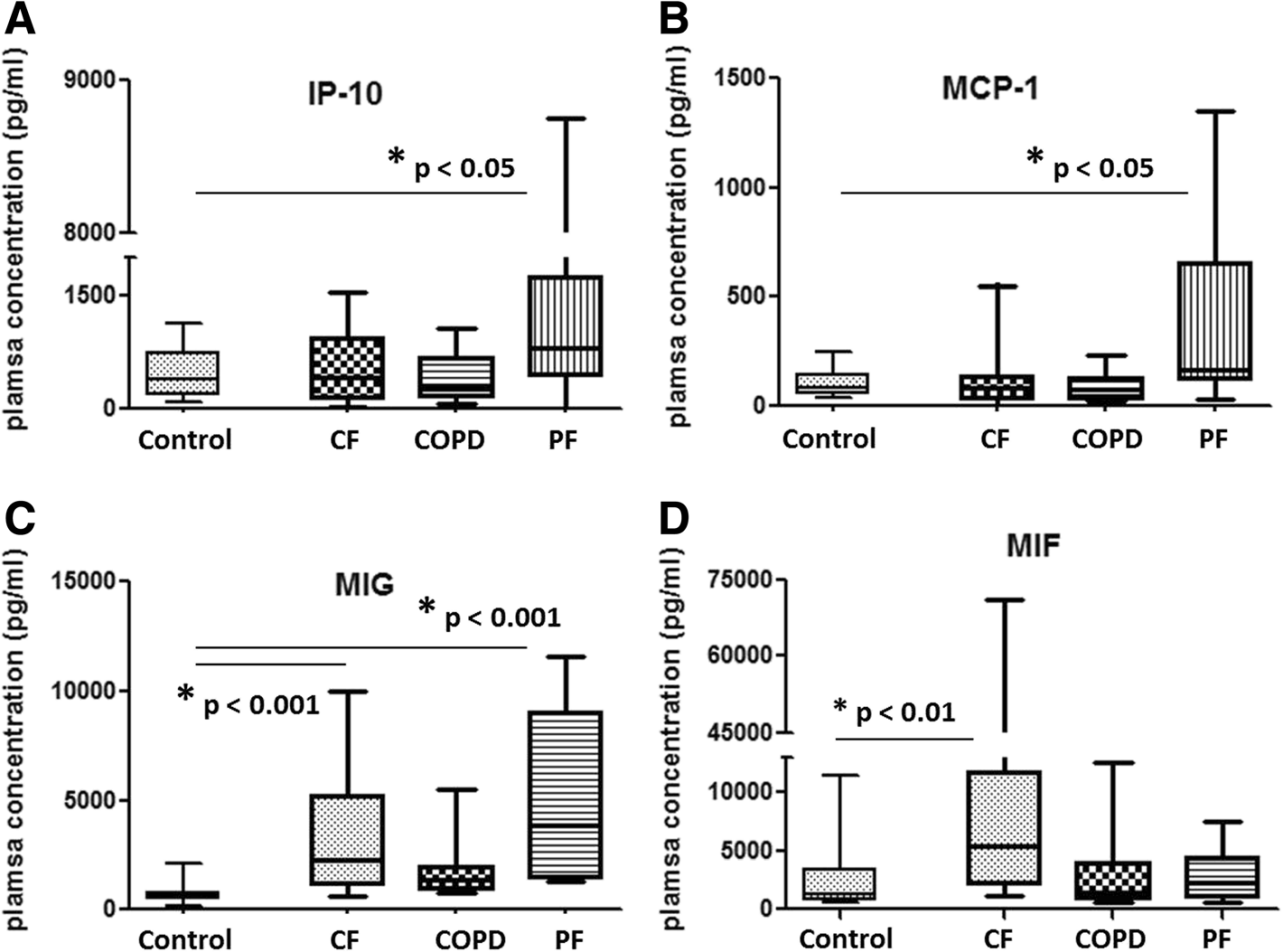
**Plasma cytokine concentrations in end-stage lung disease patients.** Comparison of plasma cytokine concentrations between cystic fibrosis (CF) (n = 19), chronic obstructive pulmonary disease (COPD) (n = 16), and pulmonary fibrosis patients (PF) (n = 17) compared to lung donor and healthy volunteer controls (n = 18). Statistically significant differences were found for **(A)** Interferon gamma-induced protein 10 (IP-10), **(B)** Monocyte Chemotactic Protein-1 (MCP-1) **(C)** Monokine-Induced by Gamma Interferon (MIG), and **(D)** Macrophage Migration Inhibitory Factor (MIF) levels. Kruskal-Wallis test with Dunn’s multiple comparison post-hoc analysis. Boxes show the median, 25th and 75th percentiles. Whiskers represent the 2.5 and 97.5 percentiles.

When the plasma protein concentrations were compared across the 3 main end-stage lung diseases, different patterns of expression were noted for some key inflammatory cytokines. Specifically, it was found that IP-10 and MCP-1 are increased in IPF patients, while MIG is increased in across all 3 end-stage groups, and MIF is specifically increased in CF patients when each were compared to lung donor and healthy volunteer control plasma (Figure 7A-D).

**Figure 8:**
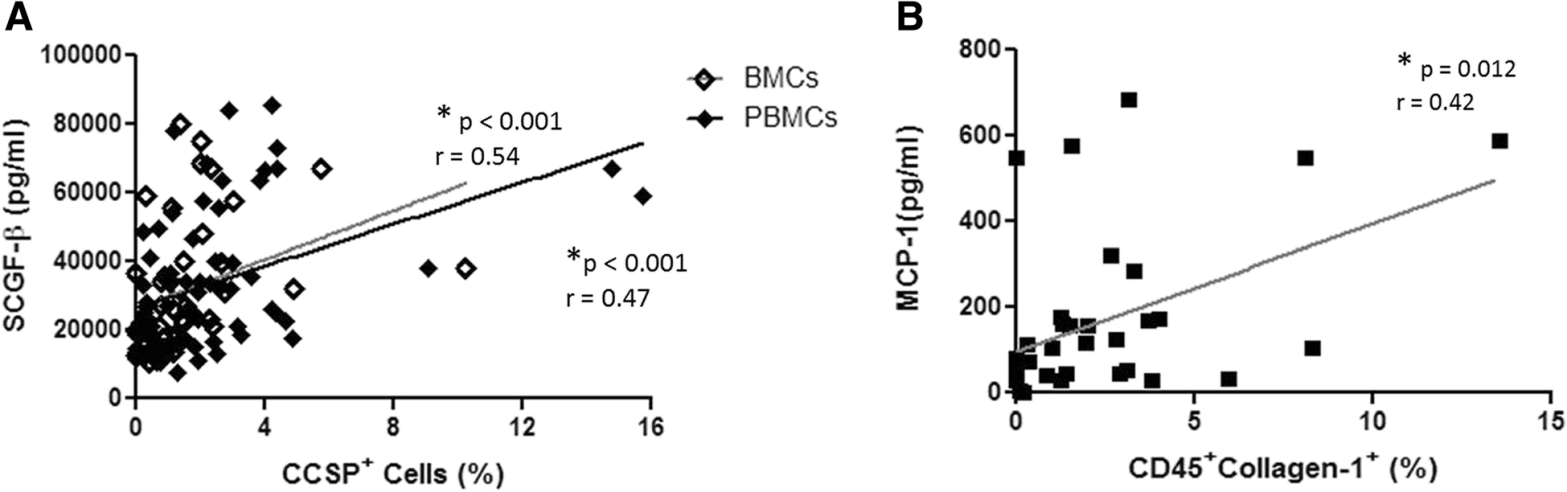
**Plasma protein concentration and progenitor cell numbers. (A)** The relationship between plasma Stem Cell Growth Factor (SCGF)-β levels and the percentage of CCSP^+^ bone marrow cells (BMCs) (n = 44) or CCSP^+^ peripheral blood mononuclear cells (PBMCs) in lung transplant recipients (R) and donors (D), and healthy controls (BMC: n = 35R, 9D. PBMC: (n = 49R, 9D, 8H). **(B)** The relationship between plasma Monocyte Chemotactic Protein (MCP)-1 levels and the percentage of CD45^+^Collagen-1^+^ fibrocytes in lung transplant recipients and donors, and healthy controls (n = 29R, 6D, 2H). Spearman rank test with correlation coefficient.

To further investigate the function of plasma protein mediators in progenitor cell recruitment, the relationship between protein concentration and cell numbers was analyzed. The number of CCSP^+^ cells in the bone marrow and peripheral blood significantly correlated with the plasma concentration of Stem Cell Growth Factor-beta SCGF-β (Figure 8A) in a range of samples, including lung transplant recipients, donors, and control samples. In addition, it was further found that fibrocyte numbers correlated with the plasma concentration of MCP-1 (Figure 8B).

## Discussion

The results presented demonstrate a relationship between the profile of putative lung progenitor cell populations and chronic lung diseases. Specific relationships between increased CCSP^+^ epithelial-like progenitors and cystic fibrosis and between increased circulating fibrocytes and fibrotic diseases such as pulmonary fibrosis and bronchiolitis obliterans were identified. Furthermore, the results suggest the involvement of key chemotactic mediators, including SDF-1, SCGF-β, and MCP-1 in the recruitment or maintenance of these cell populations within the specific disease groups studied.

In our previous publication [8] we reported that murine bone marrow contains a population of cells which express CCSP on their surface. This was confirmed by PCR on FACS-sorted populations, western blotting, and with the use of CCSP knockout mice. This population demonstrated a greater propensity to express a lung epithelial phenotype at the gene and protein level and was preferentially retained in injured lung, compared to other bone marrow cells and contributed to the epithelial lining after bone marrow transplantation. As a result of these properties, we termed these cells epithelial-like progenitor cells. We also reported that human marrow contains a similar population by flow cytometry. Here we confirm that human bone marrow and peripheral blood contain such cells using specific Taqman-based PCR probes. Of note, the amount of CCSP mRNA is roughly 60 fold lower than bronchial tissues, but many fold higher than other types of bone marrow cells.

The assessment of both epithelial-like and fibroblast progenitor cell populations in chronic end-stage lung disease patients has not been previously reported. We hypothesized that when studying such variable diseases, the measurement of two cell populations with potentially contradictory functions would provide a more complete understanding. Indeed, there are significant differences in these cell populations when compared between underlying diseases. Specifically, CCSP^+^ cells in the bone marrow and peripheral blood were increased in CF patients where small airway epithelial damage and injury may be a predominant and persisting stimulus. Acknowledging the differences between acute and chronic lung injury, and between mouse and human studies, these findings support our original observations in mice where these cells increased following an epithelial-specific naphthalene-induced airway injury. We speculate that this may be attributable to a sustained but unresolved effort to repair the damaged CF epithelium leading to a persistent inflammatory environment resulting in a perpetual recruitment signal to the bone marrow and accumulation of the CCSP^+^ epithelial-like progenitor population. It has previously been reported that bronchial epithelium from CF patients is more proliferative than that from non-CF airways [12]. Humanized airway xenografts, where CF-derived cells portrayed a greater proliferative potential, were further characterized by remodelling, delayed differentiation, and altered pro-inflammatory responses [13].

The observation that circulating fibrocytes are increased in fibrotic diseases is in agreement with prior evidence [10,14]. We also have documented that BO patients are a particularly striking subset in terms of very high numbers of fibrocytes [11]. This supports the hypothesis that circulating fibrocytes can contribute to the lung fibroblast population, either through paracrine activation of endogenous fibroblasts or by engraftment and direct contribution to matrix deposition and remodelling. The measurement of both epithelial and mesenchymal progenitor populations has identified changes in these cell numbers that correspond with changes in the underlying epithelial or mesenchymal lung pathology. Specifically, increased epithelial-like progenitors were identified in CF where the epithelium is hyperplastic, whereas mesenchymal progenitors were increased in disease characterized by fibroproliferation. Although many common mechanisms exist in end-stage lung disease patients, the unique biology of CF versus fibrotic lung disease may be further described by these novel differences in progenitor cells numbers, opening up new avenues of investigation.

Importantly, no correlations were found between patient age, gender, or BMI, suggesting that these demographic parameters do not seem to influence the observed changes in cell profiles. Yet a correlation between the proportion of CCSP^+^ BMCs and PBMCs was identified, suggesting a relationship between number of bone marrow cells in reserve and the number that can exit and traffic through peripheral blood. SCGF-β was found to correlate significantly with the number of both CCSP^+^ cell populations. It is possible that SCGF-β may act as an endogenous mitogen for the epithelial-like progenitors, as have been described for CD34^+^ hematopoietic cells [15], although the direct source of this factor has not been determined in this study. No correlations were identified between CCSP^+^ cell populations and the proportion of circulating fibrocytes. This suggests that distinct mechanisms may be responsible for the recruitment of each population and argues against a generalized alteration in marrow-derived cell mobilization or trafficking.

This study has several limitations, most importantly the cross-sectional design. Future studies will be needed to obtain data from patients at various points during the development of their lung disease. However it is doubtful that sampling of the bone marrow will be possible in such a longitudinal follow up study. In these patients, all with severe end-stage lung disease awaiting lung transplantation, there was no significant relationship between CCSP^+^ BMCs/PBMCs or CD45^+^Collagen-1^+^ cells and FEV1/FVC ratio in CF or COPD patients or with the % predicted FVC for pulmonary fibrosis patients. This does not exclude the possibility that these cell populations contribute to lung disease pathology, and further analysis in patients at much earlier stages of the lung disease will be important future priorities. In addition, many other important clinical parameters influence pulmonary function and these confounding variables may have obscured any relationship between cell numbers and lung function. Another limitation is the use of lung donors as a control group. While not ideal, as this group may well have acute or chronic damage to the lung, it represented the only option for analysis of bone marrow as sternal harvest of truly normal controls would not be ethical.

In an effort to understand the mechanisms responsible for different progenitor cell profiles between lung diseases, key chemokines and receptors were analyzed. Fibrocytes have been previously been reported to express a number of important chemokine receptors including CXCR4 [9], CCR2 [16], and CCR7 [17]. When CCSP^+^ BMCs and PBMCs were analyzed for a panel of similar receptors, expression of CCR2, CCR4, CXCR3, and CXCR4 was identified. It is expected that some pathways are redundant and some cytokines will have the ability to activate both cell populations. This is perhaps evidence of the bone marrow origin of both populations.

Migration studies for CCSP^+^ were performed to investigate the *in vitro* response to various chemokines. Migration was not analyzed for fibrocytes, as this has been previously reported [9,18]. Here, we found that Stromal Derived Factor (SDF-1) was an important migration stimulus for CCSP^+^ cells, as has also been reported for fibrocytes. It has been previously reported that neutralizing antibodies against SDF-1 can attenuate the fibrotic effects of bleomycin-induced mouse lung injury [9]. Pulmonary expression of SDF-1 has also been reported in the context of lung injury and the recruitment of bone marrow-derived cells [19]. SCGF-β was also found to induce migration of CCSP^+^ PBMCs and BMCs in end-stage lung disease patients. This supports the observed correlation between this plasma cytokine and the number of CCSP^+^ cells measured. Expression of SCGF-β transcripts is reportedly restricted to cells of the myeloid lineage [20], which may include resident lung macrophages. The expression of CCR2 by both cell populations, as well as the increase in the ligands IP-10 and MCP-1 in pulmonary fibrosis further highlights the role of inflammation in many end-stage lung diseases. The plasma concentration of MCP-1 was further shown to correlate to the number of circulating fibrocytes, again identifying a role for CCR2-mediated recruitment of progenitor cells, which may be enhanced in the fibrotic patient.

Interestingly, MIF was found to be specifically increased in CF patients, perhaps suggesting a unique role for CD74 or CXCR4 in the mechanism of CCSP^+^ cell recruitment. It has been reported that MIF can act as a ligand for CXCR4 and induce the migration of monocytes and T-cells, perhaps suggesting a novel mechanism of epithelial-like progenitor cell trafficking [21,22]. MIF is a pleiotropic inflammatory mediator with chemokine-like functions that can direct migration of leukocytes to inflammatory sites [21]. MIF has also been shown to be produced by epithelial cells [23] and activated alveolar macrophages [24], suggesting a potential mechanism by which damaged CF epithelium recruits circulating CCSP^+^ cells. Enhancing CCSP^+^ recruitment using MIF may not be a viable therapeutic option as MIF acts on multiple cells types and may exacerbate inflammatory responses.

## Conclusions

Taken together, this evidence provides new understanding of the pathogenesis of end-stage lung disease, especially cystic fibrosis. We initially hypothesized that loss of epithelial-like progenitors may be linked to impaired epithelial repair in COPD or IPF, but this was not found in this particular patient set. The association of an increase in epithelial progenitors and the changes in proliferative capacity of CF epithelium is surprising and novel. It remains to be determined if the increase in epithelial-like progenitor population in bone marrow and peripheral blood is the cause or consequence of the epithelial hyperproliferation seen in CF, which, warrants further exploration. The gain of circulating fibroblastic progenitors may contribute to the altered tissue repair and remodelling processes observed in the fibrotic lung. Alterations in circulating inflammatory and stem cell recruitment factors are likely an important element in the control of progenitor cell trafficking and in the ultimate ability to influence lung tissue repair or pathology. Modulation of important factors including MCP-1, MIF, SCGF-β and/or SDF-1 is likely an important avenue for further investigation and ultimately therapeutic intervention.

## Abbreviations

CCSP: Clara cell secretory protein; BM: Bone marrow; PBMC: Peripheral blood mononuclear cell.

## Competing interests

The authors declare that they have no competing interests.

## Authors’ contributions

*SEG*: Data acquisition. Conception, design, critical interpretation of the results. Manuscript preparation and revision. *KL*: Data Collection. Manuscript revision. *GTC*: Data acquisition, critical interpretation of the results. Manuscript revision. *MC*: critical interpretation of the study. Manuscript revision. *MS*: critical interpretation of the study. Manuscript revision. *LGS*: critical interpretation of the study. Manuscript revision. *SK*: critical interpretation of the study. Manuscript revision. *TKW*: conception, design, critical interpretation. Manuscript revision. All authors read and approved the final manuscript.

## Pre-publication history

The pre-publication history for this paper can be accessed here:

http://www.biomedcentral.com/1471-2466/13/48/prepub

## Supplementary Material

Additional file 1: Table S3 Multiplex Plasma Array Targets. This table lists the protein targets analyzed by multiplex array. Click here for file

Additional file 2: Table S1 Lung Recipient Demographics - All vs. Included. This table compares recipient demographics from patients included in this analysis compared to the demographics of all those transplanted at our centre in the same time period, indicating no significant differences in these parameters. Click here for file

Additional file 3: Table S2 Lung Donor Demographics – All vs. Included. This table compares recipient demographics from patients included in this analysis compared to the demographics of all those transplanted at our centre in the same time period, indicating no significant differences in these parameters. Click here for file

Additional file 4: Figure S1 Absolute Peripheral Blood Progenitor Cell Numbers. **(A)** Total peripheral blood leukocyte counts in end-stage lung disease patients. Normal range defined by diagnostic laboratory at the Toronto General Hospital. **(B)** Absolute CCSP^+^ cell numbers and **(C)** Absolute CD45^+^Collagen-1^+^ cell numbers calculated from leukocyte counts. Kruskal-Wallis test with Dunn’s multiple comparison post-hoc analysis. Boxes show the median, 25th and 75th percentiles. Whiskers represent the 2.5 and 97.5 percentiles. * = p < 0.05. Click here for file
