# Supplementary figures and images for: Bone marrow-derived progenitor cells in end-stage lung disease patients
Source: BMC Pulm Med. 2013 Aug 3;13:48. doi: 10.1186/1471-2466-13-48 (PMC3750607; doi:10.1186/1471-2466-13-48)

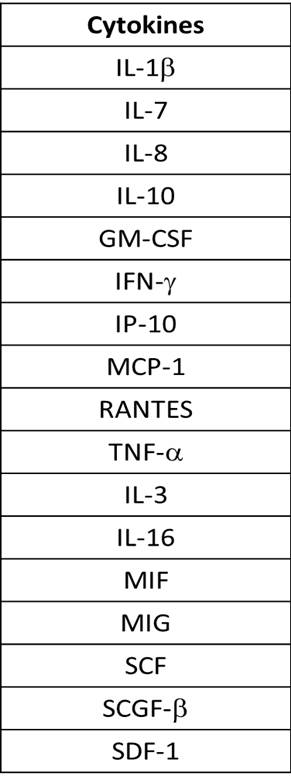

Supplement: Additional file 1: Table S3 — Multiplex Plasma Array Targets. This table lists the protein targets analyzed by multiplex array. [file 1471-2466-13-48-S1.jpeg]

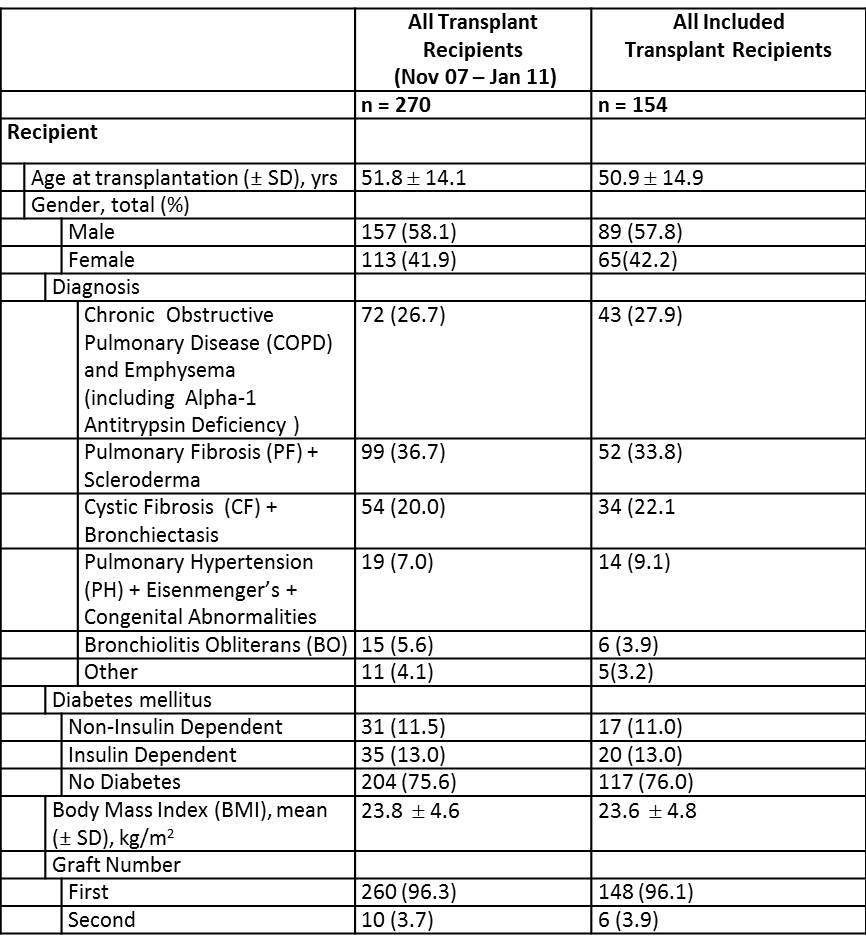

Supplement: Additional file 2: Table S1 — Lung Recipient Demographics - All vs. Included. This table compares recipient demographics from patients included in this analysis compared to the demographics of all those transplanted at our centre in the same time period, indicating no significant differences in these parameters. [file 1471-2466-13-48-S2.jpeg]

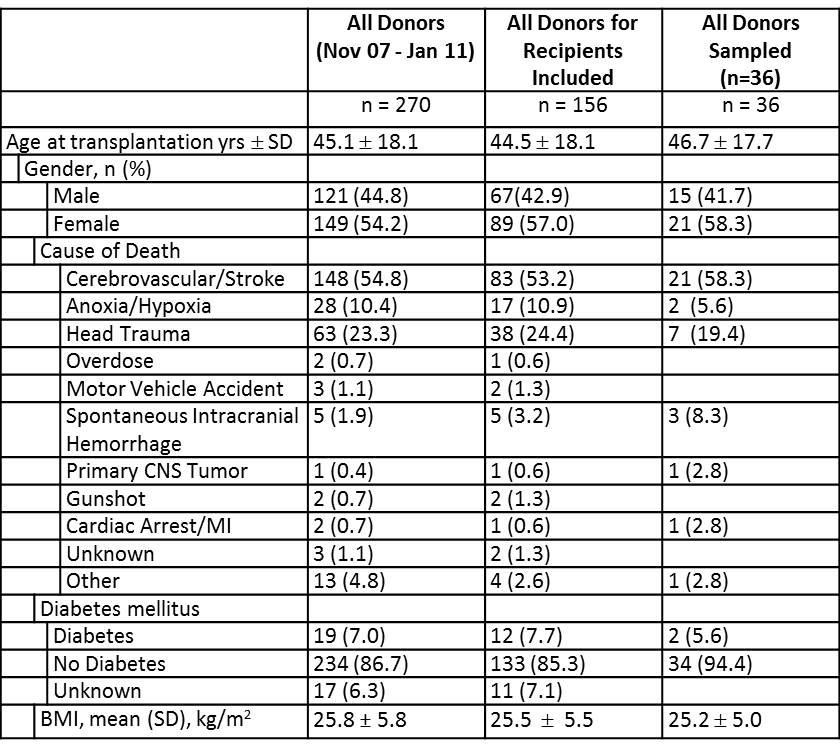

Supplement: Additional file 3: Table S2 — Lung Donor Demographics – All vs. Included. This table compares recipient demographics from patients included in this analysis compared to the demographics of all those transplanted at our centre in the same time period, indicating no significant differences in these parameters. [file 1471-2466-13-48-S3.jpeg]

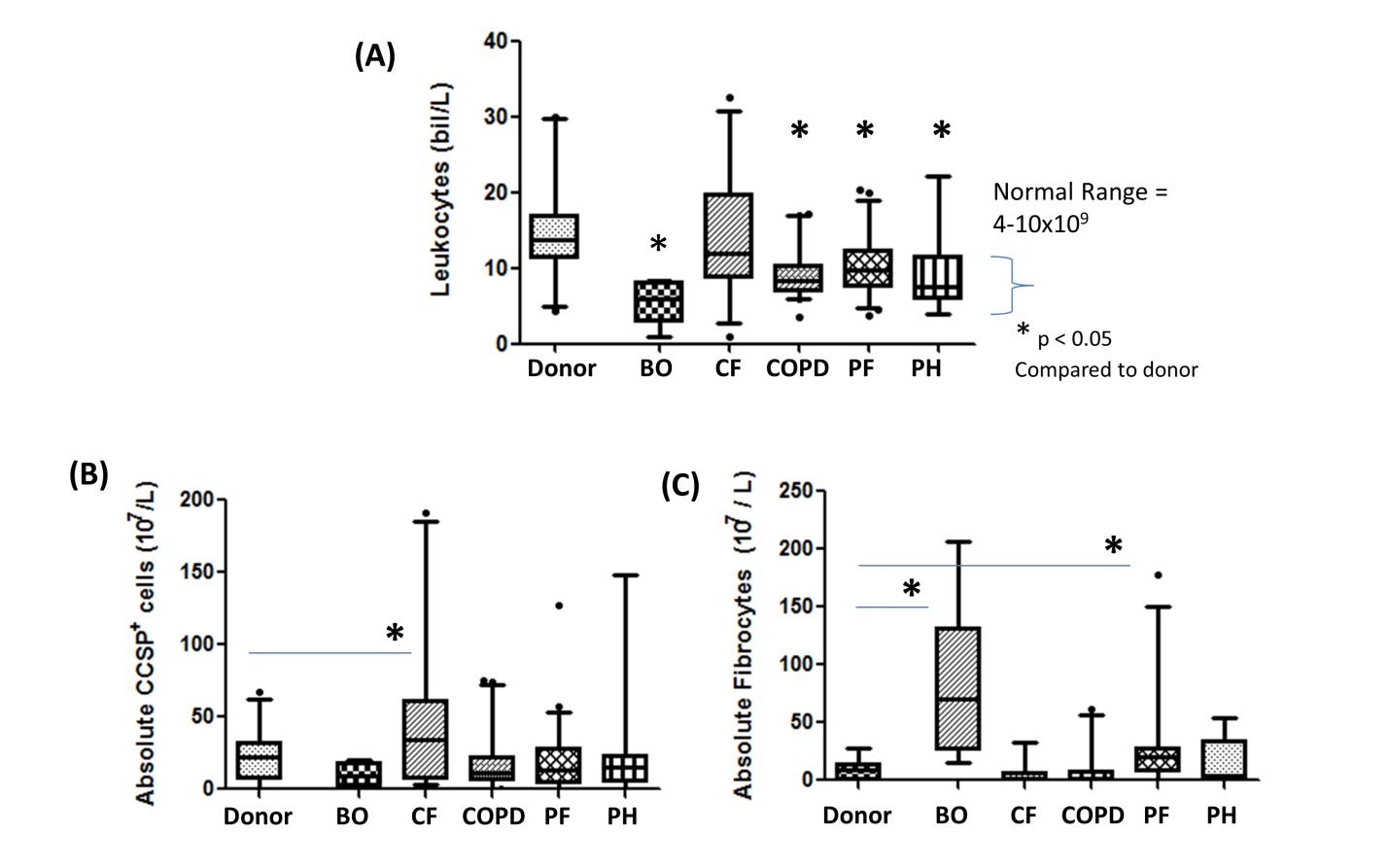

Supplement: Additional file 4: Figure S1 — Absolute Peripheral Blood Progenitor Cell Numbers. (A) Total peripheral blood leukocyte counts in end-stage lung disease patients. Normal range defined by diagnostic laboratory at the Toronto General Hospital. (B) Absolute CCSP+ cell numbers and (C) Absolute CD45+Collagen-1+ cell numbers calculated from leukocyte counts. Kruskal-Wallis test with Dunn’s multiple comparison post-hoc analysis. Boxes show the median, 25th and 75th percentiles. Whiskers represent the 2.5 and 97.5 percentiles. * = p < 0.05. [file 1471-2466-13-48-S4.jpeg]
